# Clinical outcomes of exclusive enzyme therapy (laronidase) in a cohort of patients with mucopolysaccharidosis type I

**DOI:** 10.1186/s13023-025-04157-6

**Published:** 2025-12-06

**Authors:** Nathalie Guffon, Magali Pettazzoni, Nicolas Pangaud, Nathalie Reynes, Eliane Le Peillet Feuillet, Pierre Journeau, Alain Fouilhoux

**Affiliations:** 1 Reference Center for Inherited Metabolic Disorders, Femme Mère Enfant Hospital, Hospices Civils de Lyon, Lyon, France; 2 Department of Biochemistry and Molecular Biology, Hospices Civils de Lyon, Lyon, France; 3 Cardiology, Louis Pradel Hospital, Hospices Civils de Lyon, Lyon, France; 4 Sanofi, Gentilly, France; 5 Department of Pediatric Orthopaedic and Traumatology Surgery Department, Femme Mère Enfant Hospital, Hospices Civils de Lyon, Lyon, France

**Keywords:** MPS I, Mucopolysaccharidosis type I, Laronidase, Attenuated MPS I, Severe MPS I, Enzyme replacement therapy, Hurler syndrome, Hurler-Scheie syndrome, Scheie syndrome, Glycosaminoglycan

## Abstract

**Background:**

Mucopolysaccharidosis type I (MPS I), is an autosomal recessive disorder caused by a deficiency in the enzyme α-L-iduronidase (IDUA), leading to the accumulation of glycosaminoglycans (GAGs) in tissues. Early diagnosis and treatment [i.e., bone marrow transplantation and/or enzyme replacement therapy (ERT) with laronidase] are essential to prevent irreversible damage. The long-term effectiveness of exclusive ERT has been primarily described in attenuated phenotypes, while only a few cases have been reported in severe phenotypes.

**Methods:**

This study is a retrospective analysis summarising the collective experience of disease progression in 48 patients with severe and attenuated MPS I who were treated exclusively with laronidase over a median of 10 years at the Lyon Reference Centre for Hereditary Metabolic Diseases in France. Patients were categorised by genotype and further stratified by age at treatment initiation. The study assessed the evolution of urinary excretion of GAGs, pulmonary function, cardiac involvement and evolution, height, cognitive impairment, functional status, orthopaedic and ear-nose-throat (ENT) procedures, sleep apnoea, and carpal tunnel syndrome. Descriptive statistical analysis methods were used.

**Results:**

ERT reduced the GAGus levels by 88% in severe MPS I and by 71% in attenuated MPS I, of which 47% and 65% patients, respectively achieved normal age-related GAG levels at the last follow-up. ERT provided stable or consistent improvement in forced vital capacity, slowed progression of adverse cardiac course and improved auditory transmission in majority of the severe and attenuated patients. At the last follow-up, 84% attenuated patients had normal cognitive development. In alive Hurler patients, cognitive development was very heterogenous; however, 73% patients had a developmental quotient (DQ) ≥ 70. Laronidase was effective in improving statural growth of attenuated patients treated before 9 years of age.

**Conclusion:**

Early ERT and regular multidisciplinary management are effective in slowing disease progression in severe and attenuated patients with MPS I and helping to maintain autonomy in patients with attenuated MPS I, ensuring a better quality of life.

**Supplementary information:**

The online version contains supplementary material available at 10.1186/s13023-025-04157-6.

## Introduction

Mucopolysaccharidosis type I (MPS I), is an autosomal recessive lysosomal storage disease caused by a deficiency of α-L-iduronidase (IDUA, EC 3.2.1.76) due to mutations in the *IDUA* gene, and affects ~ 1–9:1,000,000 live births [1, 2]. IDUA normally degrades the glycosaminoglycans (GAGs) heparan sulphate and dermatan sulphate. Its deficiency leads to the accumulation of GAGs in multiple tissues, resulting in a progressive and multisystemic disease [3]. MPS I is classified as severe (Hurler syndrome, OMIM#607014) with extensive multisystemic and CNS involvement, and attenuated ((Hurler/Scheie, OMIM#607015 and Scheie, OMIM#607016) with slower progression and minimal neurological involvement [1, 3–5]. Patients with Hurler syndrome typically develop coarse facial features, skeletal disease, hepatosplenomegaly, cognitive decline, hearing loss, corneal clouding, cardiac and respiratory disease with death usually before 10 years of age if untreated [1, 3, 6, 7]. Hurler-Scheie and Scheie syndromes span a wide phenotypic spectrum, with mild or no cognitive impairment, progressive somatic and skeletal manifestations that significantly impact quality of life (QoL) [1, 3, 6, 8, 9]. Timely diagnosis and prompt treatment are essential to avert the progression of severe symptoms [8].

Enzyme replacement therapy (ERT) with laronidase, a recombinant human IDUA (available since 2003), should be started as soon as possible before irreversible damage occurs [4]. Laronidase administration is safe and effective, and improvements in clinical manifestations and stabilisation of disease progression are achieved in patients with varying degrees of disease severity [3]. However, outcomes are difficult to interpret given the rarity of MPS I, its clinical variability, and limited clinical studies. The Lyon Reference Centre for Hereditary Metabolic Diseases, which has been managing MPS for more than 35 years and currently monitors around 300 patients with MPS, here reports outcomes in 48 patients with severe and attenuated MPS I treated exclusively with laronidase over a 10-year period.

## Methods

This retrospective analysis assessed data from patients enrolled in the International MPS I Registry supplemented by data from patient records. Patients with severe (Hurler syndrome) MPS I [ineligible for haematopoietic stem cell transplant (HSCT) due to unavailability of compatible donor, developmental quotient (DQ) < 70, age > 2.5 years, unfavourable cardiorespiratory condition, or splenectomy], and attenuated MPS I phenotypes (Hurler-Scheie and Scheie syndromes) treated exclusively with laronidase between 2003 (year of laronidase availability) and March 2020 were included in the analysis. Patients were categorised by severity based on genotype, and then stratified by age at treatment initiation: severe patients into ≤ 2 years vs. > 2 years; attenuated patients into ≤ 4 years, 5–9 years, and ≥ 10 years. Baseline values were defined as the closest data before and within 3 months of treatment initiation.

The study assessed the longitudinal evolution of: (1) urinary excretion of glycosaminoglycans (GAGu) hexuronic acids using harmine reagent after GAGu purification [10] expressed in µg/mg creatinine with normal values adapted to age (urine sampling was performed using the morning micturition collected immediately prior to the weekly ERT infusion), (2) pulmonary function measured by spirometry and reported as forced vital capacity percent (FVC%), (3) cardiac involvement (particularly of the aortic and mitral valves) assessed by echocardiography and electrocardiogram (ECG), (4) Z-scores for height for all patients and subgroups adjusted by age and sex, (5) cognitive function based on the age-appropriate DQ assessment tests [DQ score in patients < 6 years of age, Wechsler Intelligence Scale for Children (WISC) in patients aged 6–17 years of age, and Wechsler Adult Intelligence Scale (WAIS) for patients > 17 years of age], (6) functional status using the MPS Health Assessment Questionnaire (MPS-HAQ) questionnaires, (7) need for orthopaedic and ear-nose-throat (ENT) surgical procedures, (8) sleep apnoea syndrome (SAS) diagnosed by polysomnography, (9) hearing loss based on audiogram-and auditory evoked potentials, and (10) carpal tunnel syndrome based on electromyoneurograph (ENMG, nerve conduction test) and/or median nerve release intervention. Demographic data [mean, standard deviation, median, 25th and 75th percentiles, minimum, maximum or n (%)] were calculated for all groups/subgroups.

## Results

### Patient demographics

The study cohort consisted of 48 patients (23 patients with severe phenotype and 25 patients with attenuated phenotype).

#### Severe patient group

MPS I diagnosis was confirmed at the median age of 0.9 years. Of 23 patients, 22 received weekly laronidase infusion at the approved dose (100 U/kg/week), one discontinued early due to infusion-related reactions and was excluded from the analysis. ERT was initiated at a median of 0.1 years after diagnosis at the median age of 1.3 years. Median treatment duration was 9.6 years and median age at last follow-up was 10.6 years. Of the 22 patients, 7 (32%) died of disease-related causes at a median age of 8.9 years (5 patients from cardiorespiratory complications, and 2 during a surgical procedure); one patient died in an accident unrelated to the disease. Fourteen patients (64%; median age: 12 years) were alive at the end of follow-up, all treated at the recommended dose (100 U/kg/week) with a median duration of 11.5 years. Demographic characteristics are provided in Table 1.

**Table 1:** Demographics, clinical and genotypic characteristics of patients with severe MPS I treated with laronidase

	Total	Age at ERT initiation	
Parameters	Severe	≤ 2 years	>2 years
**Total number of patients (N)**	23	16	7
**Genetic variants on ***IDUA ***gene **(NM_000203.5)			
p.W402X/p.W402X	4	4	0
p.Q70X/p.Q70X	2	2	0
p.Q70X/p.W402X	2	1	1
p.P533R/p.P533R	2	1	1
p.W402X/p.A327P	4	3	1
p.W402X/p.P533R	1	0	1
p.W402X/c.206del	1	1	0
c.35_46del/c.1524 + 1 G > A	1	1	0
c.651_667del/c.651_667del	1	0	1
c.1650 + 5 G > A/c.1650 + 5 G > A	1	0	1
c.1828 + 1 G > A/c.1828 + 1 G > A	1	1	0
p.Y343X/p.Q70X	1	0	1
p.W47X/p.D413_L421del	1	1	0
p.E123X/c.1589_1596dup	1	1	0
**Age at diagnosis (years)**			
n	23	16	7
Mean (SD)	1.3 (0.83)	0.8 (0.45)	2.3 (0.43)
Median (25th, 75th)	0.9 (0.6, 2.0)	0.8 (0.6, 0.9)	2.1 (2.0, 3.0)
Min, max	0.0, 3.0	0.0, 1.7	2.0, 3.0
**Diagnosis period**			
Before 2003, n (%)	5 (21.7)	3 (18.8)	2 (28.6)
2003–2010, n (%)	13 (56.5)	9 (56.3)	4 (57.1)
After 2010, n (%)	5 (21.7)	4 (25.0)	1 (14.3)
**Age at initiation of ERT (years)**			
n	23	16	7
Mean (SD)	1.6 (0.92)	1.1 (0.50)	2.7 (0.52)
Median (25th, 75th)	1.3 (0.9, 2.2)	1.0 (0.8, 1.4)	2.5 (2.2, 3.1)
Min, max	0.0, 3.5	0.0, 1.9	2.2, 3.5
**Period at initiation of ERT**			
Before 2003, n (%)	0	0	0
2003–2010, n (%)	18 (78.3)	12 (75.0)	6 (85.7)
After 2010, n (%)	5 (21.7)	4 (25.0)	1 (14.3)
**Duration between diagnosis and initiation of ERT (years)**			
n	23	16	7
Mean (SD)	0.3 (0.32)	0.2 (0.25)	0.4 (0.44)
Median (25th, 75th)	0.1 (0.1, 0.4)	0.1 (0.1, 0.4)	0.2 (0.1, 0.4)
Min, max	0.0, 1.3	0.0, 0.8	0.1, 1.3
**Age at last follow-up (years)**			
n	23	16	7
Mean (SD)	10.0 (5.37)	8.8 (5.29)	12.6 (4.90)
Median (25th, 75th)	10.6 (4.5, 14.9)	8.9 (3.7, 13.6)	12.2 (8.8, 16.1)
Min, max	1.1, 19.1	1.1, 16.2	4.4, 19.1
**Total duration of follow-up since initiation of ERT (years)**			
n	23	16	7
Mean (SD)	8.4 (5.22)	7.7 (5.24)	9.9 (5.23)
Median (25th, 75th)	9.6 (3.0, 13.0)	7.8 (2.1, 12.5)	10.1 (5.7, 13.7)
Min, max	0.3, 16.6	0.3, 15.0	0.9, 16.6
**Death, n (%)**	8 (34.8)	6 (37.5)	2 (28.6)
**Age at death (years)**			
n	8	6	2
Mean (SD)	8.8 (4.94)	8.4 (4.41)	10.2 (8.28)
Median (25th, 75th)	8.9 (4.4, 12.7)	8.9 (4.5, 10.6)	10.2 (4.4, 16.1)
Min, max	2.5, 16.1	2.5, 14.9	4.4, 16.1

ERT, enzyme replacement therapy; MPS I, mucopolysaccharidosis type I; SD, standard deviation

#### Attenuated patient group

The 25 attenuated patients, 22 Hurler-Scheie syndrome and 3 Scheie syndrome, were diagnosed at a median age of 4.2 years. ERT was initiated at a median of 0.6 years after diagnosis at median age of 6.7 years. All patients received a weekly laronidase infusion of 100 U/kg/week. Median treatment duration was 9.7 years and median age at last follow-up 17.7 years. Five patients (4 Hurler-Scheie and 1 Scheie) who started ERT in adulthood (median age of 17.7 years) died at a median age of 17.7 years: 3 deaths were disease-related: (1 after spinal surgery, 1 failed tracheal intubation for cardiac valve replacement surgery, and 1 arrythmia 4 years after ERT discontinuation) and 2 unrelated (1 accident and 1 lung cancer from smoking). Demographic characteristics are provided in Table 2.

**Table 2:** Demographics, clinical and genotypic characteristics of patients with attenuated MPS I treated with laronidase

	Total	Age at ERT initiation		
Parameters	Mitigated	≤ 4 years	5–9 years	≥10 years
**Total number of patients (N)**	25	7	7	11
**Genetic variants on ***IDUA ***gene **(NM_000203.5)				
p.P533R/p.P533R	5	1	2	2
p.W402X/p.P533R	2	2	0	0
c.46_57del/p.S633L	2	2	0	0
p.P533R/p.S633L	3	1	1	1
p.E276K/del exons 1 and 2	2	0	2	0
p.W402X/p.L238P	1	0	0	1
p.W402X/p.R89Q	1	0	0	1
p.W402X/p.C577Y	1	0	0	1
p.W402X/ p.D203N	1	1	0	0
p.L209R/p.L209R	1	0	1	0
p.Q70X/p.P533R	1	0	0	1
p.S586F/p.S586F	1	0	0	1
p.Q70X/p.D203H	1	0	0	1
p.E178K/c.46_57del	1	0	0	1
p.L18P/p.P496R	1	0	1	0
Unknown	1	0	0	1
**Age at diagnosis (years)**				
n	25	7	7	11
Mean (SD)	7.7 (9.70)	2.2 (0.96)	4.9 (2.40)	13.0 (12.84)
Median (25th, 75th)	4.2 (2.4, 8.0)	2.4 (1.4, 3.1)	4.2 (4.0, 6.7)	8.0 (4.7, 20.2)
Min, max	0.8, 39.7	0.8, 3.2	1.0, 8.6	1.2, 39.7
**Diagnosis period**				
Before 2003, n (%)	8 (32.0)	0	1 (14.3)	7 (63.6)
2003–2010, n (%)	11 (44.0)	3 (42.9)	4 (57.1)	4 (36.4)
After 2010, n (%)	6 (24.0)	4 (57.1)	2 (28.6)	0
**Age at ERT initiation (years)**				
n	25	7	7	11
Mean (SD)	13.5 (12.98)	2.4 (0.94)	5.7 (1.71)	25.4 (10.78)
Median (25th, 75th)	6.7 (3.3, 22.0)	2.8 (1.5, 3.2)	5.6 (4.1, 6.7)	24.4 (14.7, 36.3)
Min, max	1.2, 41.3	1.2, 3.3	4.1, 8.7	10.8, 41.3
**Period at initiation of ERT**				
Before 2003, n (%)	5 (20.0)	0	1 (14.3)	4 (36.4)
2003–2010, n (%)	12 (48.0)	3 (42.9)	4 (57.1)	5 (45.5)
After 2010, n (%)	8 (32.0)	4 (57.1)	2 (28.6)	2 (18.2)
**Duration between diagnosis and initiation of ERT (years)**				
n	25	7	7	11
Mean (SD)	5.8 (9.84)	0.2 (0.16)	0.8 (1.70)	12.5 (11.91)
Median (25th, 75th)	0.6 (0.1, 5.7)	0.1 (0.0, 0.4)	0.1 (0.0, 0.6)	9.1 (1.6, 23.3)
Min, max	0.0, 31.6	0.0, 0.4	0.0, 4.6	0.6, 31.6
**Age at last follow-up (years)**				
n	25	7	7	11
Mean (SD)	23.3 (15.13)	10.0 (4.72)	16.1 (3.99)	36.4 (13.33)
Median (25th, 75th)	17.7 (13.1, 39.1)	10.1 (6.3, 13.1)	16.0 (14.1, 18.1)	42.4 (20.6, 46.1)
Min, max	2.3, 51.0	2.3, 16.4	10.1, 22.8	17.7, 51.0
**Total duration of follow-up since initiation of ERT (years)**				
n	25	7	7	11
Mean (SD)	9.9 (4.60)	7.6 (3.99)	10.4 (3.92)	11.0 (5.19)
Median (25th, 75th)	9.7 (7.3, 13.3)	7.4 (5.2, 9.8)	9.9 (7.3, 13.3)	9.7 (6.9, 15.7)
Min, max	0.7, 17.9	0.7, 13.3	6.0, 17.2	3.0, 17.9
**Death, n (%)**	5 (20.0)	1 (14.3)	1 (14.3)	3 (27.3)
**Age at death (years)**				
n	5	1	1	3
Mean (SD)	27.7 (19.59)	6.3 (-)	17.6 (-)	38.2 (17.96)
Median (25th, 75th)	17.7 (17.6, 46.0)	6.3 (6.3, 6.3)	17.6 (17.6, 17.6)	46.0 (17.7, 51.0)
Min, max	6.3, 51.0	6.3, 6.3	17.6, 17.6	17.7, 51.0

ERT, enzyme replacement therapy; MPS I, mucopolysaccharidosis type I; SD, standard deviation

### Evolution of GAGu

#### Severe patient group

**Fig. 1:**
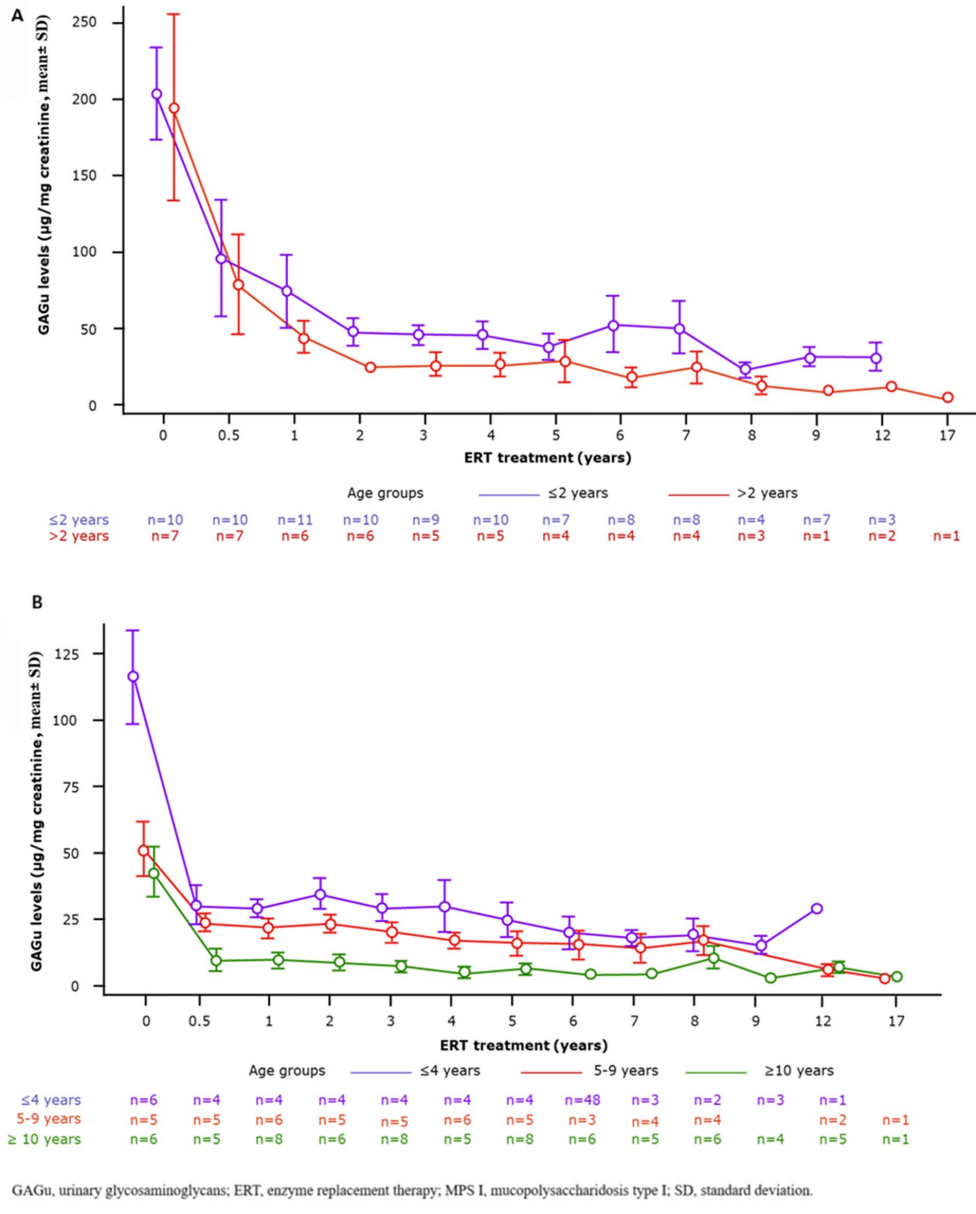
GAGu levels changes in (**A**) severe and (**B**) attenuated patients with MPS I by subgroup

Baseline GAGu data were available for 17/22 (77%) patients; missing data were due to assay changes in 3 patients or diagnosis at another site in 2 patients. The mean baseline GAGu value was 199.8 μg/mg creatinine (7 times the normal value for age; Supplementary Table 1). GAGu levels significantly reduced by 55.3% at 6 months (3 times the normal value for age), 68% at 1 year (2.8 times the normal value for age), 80.4% at 2 years (1.7 times the normal value for age), and 88% at 9 years of treatment (1.5 times the normal value for age; Fig. 1A). Significant reduction occurred within the first 6 months, continued over 2 years, and stabilised up to 10 years of follow-up, with similar trends in both age subgroups. At last follow-up, 8/17 patients (47%) had normal GAGu levels for age.

#### Attenuated patient group

Baseline GAGu data were available for 17/25 (68%) patients; data from 8 patients were excluded due to diagnoses at another site with noncomparable assays. GAGu levels significantly reduced by 70.8% at 6 months, 74.2% at 1 year, and 71.2% at 2 years, and stabilised over a median duration of 10 years at about 1.5 times the upper normal value for age. Trends were consistent across all three age subgroups (Fig. 1B). At last follow-up, 11/17 patients (65%) had normal GAGu levels for age.

### Pulmonary function/evolution of FVC% predicted

#### Severe patient group

**Fig. 2:**
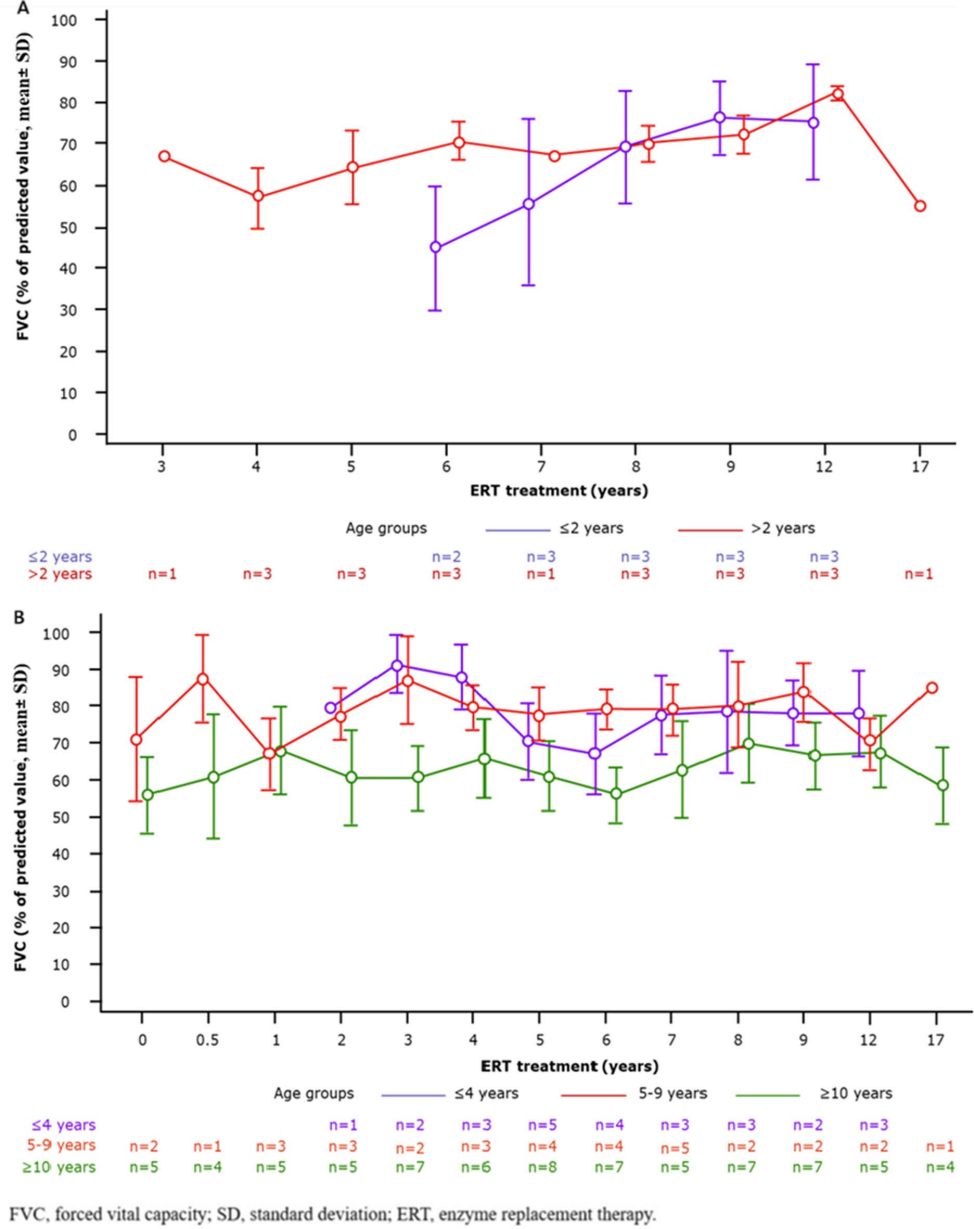
FVC progression changes in (**A**) severe and (**B**) attenuated patients with MPS I by subgroup

FVC% data were available for only 7 patients due to lack of patient cooperation or inability to perform the test reliably. At the last follow-up 10–14 years after ERT initiation, the mean FVC (± SD) was 79% (± 6.55). At the last follow-up, FVC remained stable at 99%, 55% and 72% for 3 patients treated before 2 years of age. Of the 4 patients treated after 2 years, 3 improved to normal FVC (83%, 80%, 85%), while 1 remained at 55% after 17 years. (Fig. 2A).

#### Attenuated patient group

FVC data were available for 20/25 (80%) patients (Fig. 2B). Mean FVC (± SD) improved from 60.4% (± 8.61) prior to ERT initiation to 71.2% (± 5.78) after 10 years; 15 patients (75%) showed either improved or stable FVC over 4–17 years (median: 10 years). Five patients (25%) deteriorated (from 44–94% to 32–70%) over 4–17 years (median: 7 years); 4 had high anti-laronidase antibody levels, and 1 had late treatment initiation at 36 years of age. Two patients died of disease-related complications at 18 and 46 years of age.

### Patients with sleep apnoea syndrome

#### Severe patient group

Before ERT, only one (4%) patient treated before the age of 2 years had SAS without respiratory assistance at the age of 0.6 years; this patient died at 4.5 years due to cardiorespiratory complication. After ERT (median: 7.5 years, 2–15 years), 6 (27%) patients treated before the age of 2 years developed SAS; 2 died at 9 years of age from cardiorespiratory complications, and 2 required non-invasive ventilation. SAS was not reported in 15/22 (68%) severe patients.

#### Attenuated patient group

Before ERT, 5/25 (20%) attenuated patients had SAS (1 before age 4 years and 4 after age 10 years). Two patients died at 17.7 and 46 years (the latter 4 years after stopping ERT). After treatment, 3 patients (15%) developed SAS at 2, 1.5, and 16 years after treatment initiation, at ages 2.7, 7, and 38 years. One child died accidentally from asphyxia due to a hard candy false route at age 6 years. Overall, 17/25 (68%) patients did not have SAS at last follow-up.

### Evolution of cardiac impairment

#### Severe patient group

**Fig. 3:**
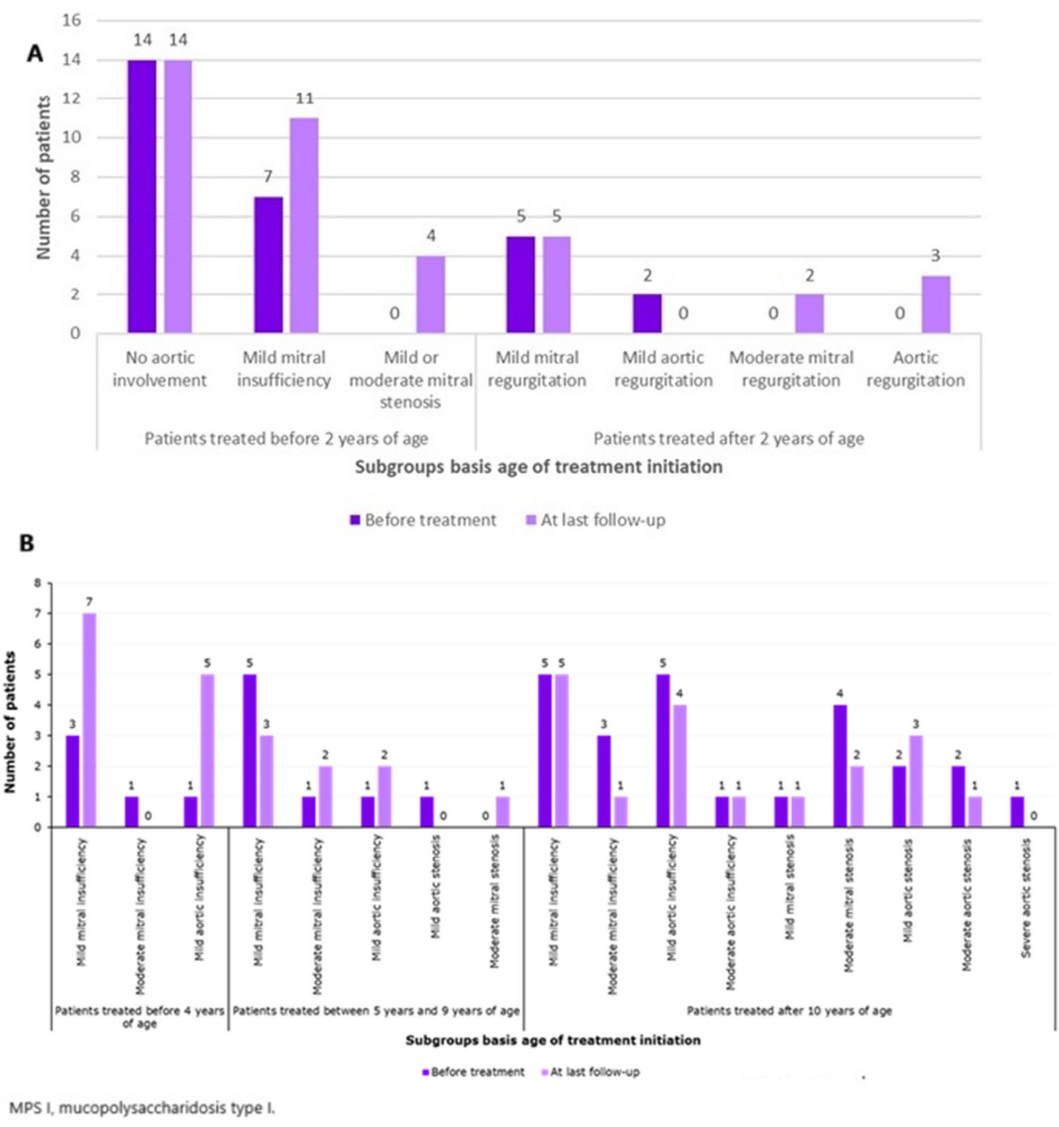
Cardiac impairment in (**A**) severe and (**B**) attenuated MPS I patients before and after treatment

Before ERT, 18/22 (82%) patients had thickened mitral and /or aortic valvular dystrophy at a median age of 1.3 years. Fourteen (63%) had mitral regurgitation [mild in 13 (93%) and moderate in 1 (7%)], 1 (5%) patient had mild aortic regurgitation, 1 (5%) had mild aortic and mitral regurgitation. No patient had mitral stenosis or aortic stenosis. At the last follow-up (median 8 years of ERT), 9/22 (41%) patients had stable valvular involvement, 5 (23%) developed mild mitral regurgitation, 4 (18%) developed mild aortic regurgitation, 5 (23%) developed mild mitral stenosis, and none of the patients developed aortic stenosis (Fig. 3A).

#### Attenuated patient group

Before ERT, all 25 patients (100%) had thick mitral valve and 24 (96%) also had thick aortic valve. Two (8%) patients had undergone mitral and aortic valve replacement at 28 and 36 years of age. Of the other 23 patients, 18 (78%) had mitral regurgitation (14 mild and 4 moderate), 8 (35%) had aortic regurgitation (7 mild and 1 moderate), 6 (26%) had aortic stenosis (4 mild and 2 moderate) and 5 (22%) had mitral stenosis (1 mild and 4 moderate). At the last follow-up (median 10.5 years of ERT), 4/23 (17%) patients underwent valve replacement. Of the 19 remaining patients, all (100%) had mitral regurgitation (17 mild and 2 moderate), 11 (58%) had aortic regurgitation (10 mild and 1 moderate), 7 (37%) had mitral stenosis (3 mild, 3 moderate, 1 severe), and 5 (26%) had aortic stenosis (4 mild and 1 moderate, Fig. 3B). Treatment initiation prevented the valvulopathy progression in most patients. Overall, mitral valvulopathy stabilised in 66% patients, and aortic valvulopathy in 63% patients after ~ 10 years of treatment. The functioning of the valve prostheses was satisfactory in the five operated patients.

### Evolution of growth

#### Severe patient group

Height data were available for 17/22 (74%) patients (13 treated before the age of 2 years and 4 treated after the age of 2 years). The height of patients was in the normal range at ERT initiation with a mean Z-score (± SD) of 0.1 (0.4). A progressive decline in height Z-score was observed from 6 months after treatment initiation: mean Z-score − 0.1 at 6 months, − 0.4 at 1 year, − 1.1 at 2 years, − 1.7 at 3 years, − 2.5 at 5 years, − 3.1 at 8 years, − 3.7 at 9 years, and  − 4.1 at 10–14 years. (Supplementary Figure 1A).

#### Attenuated patient group

Height data were available for 17/25 (68%) patients (6 treated before the age of 4 years, 4 between 5 and 9 years, and 7 after the age of 10 years). For the subgroup with ERT initiation before the age of 4 years, height was normal at baseline with a mean Z-score of − 0.5, followed by a very slow decline in growth velocity with a mean Z-score of − 1.9 after 10 to 14 years of treatment. For the subgroup with ERT initiation between the ages of 5 and 9 years, height at baseline was normal with a mean Z-score of + 0.2, followed by a very slight and slow decrease in growth velocity with a Z-score of − 1.8 after 8 years of treatment. Beyond that, growth improved, probably due to a better pubertal peak than in the natural history of the disease, with a mean Z-score of − 0.6 after 10 to 14 years of treatment. The growth deficiency remained stable in patients treated after 10 years of age (mean Z-score of − 3 before ERT initiation to a mean Z-score of − 2.7 after 10 to 14 years of treatment (Supplementary Figure 1B).

### Evolution of cognitive development

#### Severe patient group

Fourteen (64%) patients were alive at the last follow-up. Three of them were under 2 years of age at the last follow-up and had DQ < 70; in the 2 patients with follow-up data available, DQ improved from 44 to 64 in 1 patient and decreased from 65 to 53 in the other patient after 6 months of treatment. The remaining 11 alive patients had ERT initiation at a median age of 1.6 years, and the median duration of treatment at the last follow-up was 13 years. Before ERT, 3/11 (27%) had severe cognitive impairment, with DQ of 62 in 1 patient and 50 in 2 patients. Post-treatment, 1 patient remained stable (DQ = 56) after 17 years, 1 showed initial improvement (DQ = 83 at 3 years), followed by decline and stabilisation at intellectual quotient (IQ) = 70 up to 11 years, and the third patient improved to a normal IQ of 90 after 15 years. One patient with a slightly lower than normal DQ of 79 before treatment initiation improved until reaching a DQ of 90–100 during the first 4 years and then deteriorated after 5–6 years to stabilise at IQ = 70 until the last follow-up at 12 years of ERT. Seven patients had a normal DQ > 80 at baseline. One patient (DQ = 81 at baseline) progressed to a normal DQ of 92 during the first 4 years of treatment and deteriorated sharply thereafter (IQ < 30) due to disease-related sensory (visual and auditory) and motor losses. One patient (DQ = 81 at baseline) deteriorated slightly with DQ = 74 at 1 year, and then remained stable at DQ = 75 during the following 4 years of ERT. One patient (normal DQ of 100 at baseline) gradually regressed until reaching an IQ of 39 after 7 years of treatment. One patient (normal DQ of 93 at baseline) remained stable for the first 3 years of ERT and then regressed to a stable IQ of 75 for 5 years. The remaining three patients maintained a normal IQ of 93, 100, and 100 after 12, 9, and 5 years of treatment (Supplementary Figure 2).

For the eight patients who died at a median age of 9 years, ERT was initiated at a median age of 1.2 years between 2003 and 2006. The mean DQ was 62 (median 60) at baseline. In these patients, DQ progressed from 6 months up to 2–3 years of ERT (DQ = 79), followed by a decline from 4 years of ERT (IQ = 54) until the patients’ death.

#### Attenuated patient group

Twenty-one (84%) of the 25 patients had normal cognitive development, and none of the patients developed cognitive regression. Before ERT, 4 (16%) patients had cognitive impairment since the median age of 7.3 years. One patient with severe Hurler-Scheie phenotype (DQ = 50 at baseline at the age of 14.7 years) died at 17.7 years due to a postoperative neurological complication from spinal surgery. The test was no longer feasible in one patient aged 19.5 years due to severe hearing and visual impairment. Two patients aged 19 and 46 years remained stable with professional activity in a protected environment (IQ between 60 and 70).

### Functional status of patients at the last follow-up (MPS-HAQ)

#### Severe patient group

The data on the MPS-HAQ questionnaire at the last follow-up was available in 11 patients **(**Supplementary Table 2). Seven patients aged 6–16 years had a normal to subnormal autonomy of their daily activities between 0 and 2.04 out of 10, a competency score between 78% and 100%, a normal to subnormal mobility score between 0 and 2.9 out of 10, a mobility skill score of 100%, and a very low assistance score between 0 and 6 out of 39. One patient aged 12 years had moderate autonomy in daily activities (score 2.44), an autonomy skill score of 63%, a mobility score of 8, a mobility skill score of 100%, and required moderate assistance (score 27). Two patients aged 13 and 19 years had a total lack of autonomy requiring complete assistance (assistance score 39 and 30, respectively). One patient aged 8 years had moderate autonomy in daily activities (score 2.48), an autonomy skill score of 33% requiring moderate assistance (score 16); normal mobility (mobility score of 0.3 and a mobility competence score of 100%). Autonomy and mobility were good for 7/11 (64%) patients.

#### Attenuated patient group

The data on the MPS-HAQ of 16 patients were available at the last follow-up (Supplementary Table 3). Five patients had a total or subtotal autonomy with a score between 0 and 3.44, a 100% autonomy competence score, a normal or subnormal mobility score between 0 and 3.5 and a mobility competence score of 100% and required minimal assistance (score between 0 and 4). One patient treated since the age of 22 years had moderate autonomy (autonomy score 4.2 and competence score 100%) and low mobility (mobility score 6.9 and competence score 100%) and required moderate assistance with a score of 15. One patient treated since the age of 36 years lacked self-care autonomy (autonomy score 4.67 and autonomy competence score 11%) and mobility (mobility competence score 0%), requiring total assistance (score 36) due to major osteoarticular involvement. Four patients with treatment initiation between 5 and 9 years of age had normal autonomy (autonomy score between 0 and 2.3, autonomy competence score of 100%), normal to subnormal mobility with a score between 0 and 3.8 and a mobility competence score of 100% and required minimal or no assistance (score between 0 and 9). Five patients with ERT initiation before 4 years of age had normal autonomy (autonomy score between 0 and 1.3, autonomy competence score of 100%), normal to subnormal mobility with a score between 0 and 3.5 and a competence score of 100% and required minimal or no assistance (maximum score 6).

### Orthopaedic and ENT surgical procedures

#### Severe patient group

Before ERT, 2/22 (9%) patients had an orthopaedic spinal surgery and a tenotomy at the age of 1.5 and 3.5 years. 11/22 (50%) patients had one or more ENT procedures (6 adenoidectomy, 3 tonsillectomy and11 T-tube insertion) at a median age of 1.3 years. During treatment, 14/22 (64%) patients had an orthopaedic surgery (hip, spine and knee), and 14/22 (64%) had an ENT intervention at a median age of 2.7 years (13 adenoidectomy, 9 tonsillectomy and 13 T-tube insertion). After 3–4 years of treatment, the ENT condition of patients improved, and the need of ENT surgery became exceptional.

#### Attenuated patient group

Before ERT, 7/25 (28%) patients had orthopaedic surgery at a median age of 4.5 years (hip, knee, spinal cord decompression, and tenotomy), and 13/25 (52%) had an ENT procedure at the median age of 2.2 years (11 adenoidectomy, 6 tonsillectomy, and 9 T-tube insertion). During treatment, 12/25 (48%) had orthopaedic surgery at a median age of 10 years (hip, knee, spinal cord decompression, and tenotomy), and 9/25 (36%) patients had an ENT intervention at a median age of 4.7 years (4 adenoidectomy, 4 tonsillectomy and 8 T-tube insertion). After 3–4 years of ERT, the ENT status of attenuated patients improved and the need for ENT surgery became exceptional.

### Patients with hearing impairment

#### Severe patient group

Before ERT, 12/22 (54%) patients had hearing impairment at a median age of 1.3 years (9 with ERT initiation before 2 years, and 3 after 2 years; the median age of onset was 1.1 years and 2.4 years, respectively). At the last follow-up, 6/10 (60%) patients developed hearing impairment at a median age of 4.9 years (3 each with ERT initiation before 2 years and after 2 years of age; the median age of onset was 3 years and 3.5 years, respectively). At the last follow-up, 71% of alive severe patients (median age 11.1 years) had hearing impairment, with 50% using hearing aids.

#### Attenuated patient group

Before ERT, 6/25 (24%) patients had hearing impairment at a median age of 3.9 years (median age in the 3 subgroups were 1.8, 3.9 and 20.2 years, respectively). At the last follow-up, 8/19 (42 %) developed hearing impairment at a median age of 10.4 years (4 each with ERT initiation before 4 years and after 10 years of age; the median age of onset was 5 and 32.5 years, respectively); 2 patients used hearing aids since 2.7 and 6 years of age. At the last follow-up, 50% of alive attenuated patients (median age 18 years) had hearing impairment, 2 required hearing aids.

### Patients with carpal tunnel syndrome

#### Severe patient group

Before ERT, 6/22 (27%) patients had carpal tunnel syndrome at a median age of 2.3 years. At last follow-up, 10/16 (62% 45%) patients developed carpal tunnel syndrome at a median age of 4 years. Overall, 16/22 (73%) severe patients underwent carpal tunnel surgery between the ages of 2.2 years and 11 years, either before or after ERT.

#### Attenuated patient group

Before ERT, 15/25 patients (60%) had carpal tunnel syndrome at a median age of 9.2 years. At the last follow-up, 8/10 (80%) developed carpal tunnel syndrome at a median age of 9.2 years. In the three subgroups, the median age at diagnosis was 3.6, 13.2, and 40.3 years, respectively. Overall, 22/25 (88%) patients underwent median nerve release surgery.

## Discussion

### Severe patient group

The present analysis confirmed that ERT with laronidase improves survival in patients with severe (Hurler syndrome) MPS I. Mortality associated with disease is 32% after a median follow-up of 9.6 years, compared with a natural history death rate of 83% at 10 years [10]. The median age of death in the present cohort of patients with severe Hurler MPS I was 9 years, consistent with the findings of the study by Eisengart et al. (2018) on an international cohort of patients treated with laronidase, which reported a median age of death of 6.4 years without treatment [10]. In the present study, the reduction in the GAGu levels exceeded 55% after 6 months, and was 80% after 2 years; 88% of patients remained stable over the entire treatment period of ~ 9 years, and 47% had GAGu levels within the normal range for age at the last follow-up. Jones et al. (2020) showed that the efficacy of ERT is notable with a ≥ 40% reduction in GAGu levels within 6 months of ERT initiation and was sustained throughout a mean treatment duration of 6.8 years. Reduction in the GAGu levels of 50%–70% were associated with improvements in 6-minute walk test, growth rate, and the Clinician Global Impression of Change (CGI-C) score [11]. Studies by Wraith et al. [12], Clarke et al. [13] and Kakkis et al. [14] have shown mean reduction of GAGu levels by 61.3%, 72% and 63%, respectively. In the current analysis, all patients with available data showed stable or consistent improvement in FVC%, after a median treatment period of 12.5 years, with the longest duration being 17.5 years. Notably, a study by Wraith et al. (2004) also reported significant improvement in FVC% by 4.9 points within 26 weeks of treatment [15]. Jones et al. (2020) also reported significant improvement in FVC% in patients with normal or nearly normal GAGu levels [11]. In another study by Guffon et al. (2021), patients with Hurler syndrome reported impaired but stable pulmonary function 20 years post-HSCT [16]. ERT slowed down the adverse cardiac course, with about half of the patients having stable valvular involvement. In patients with severe MPS I (median age of 1.3 years), height was slightly above the 50th percentile (mean Z-score of 0.1 (0.4) before treatment initiation. This is in agreement with a study by Viskochil et al. (2019), which reported that in severe cases, growth typically followed reference curves between 12 and 24 months of age, then declined below the third percentile (Z-score = − 2) by 4 years of age, underscoring the natural history in severe patients [17]. Regardless of the age of ERT initiation before or after 2 years of age, growth progressively declined. In the total group, the mean Z-score was − 3.1 after 8 years of treatment, corresponding to 118 cm at ~ 10 years of age. These findings indicated an improvement in the growth with ERT compared with the natural history of patients with Hurler syndrome described by Viskochil et al. (2019) from the Registry data and Neufeld and Muenzer (2019), with a maximal height of 110 cm without treatment [16, 18–20]. However, it is important to note that in the present study, after 8 years of treatment, the growth deficit was less severe in patients with treatment initiation after 2 years of age (mean Z-score = − 1 versus − 4.5 for patients with treatment initiation before 2 years of age), which could be related to the severity of the skeletal involvement. The cognitive development of the 11 alive patients at the end of follow-up was very heterogeneous. These heterogeneous findings echo the varied neurodevelopmental outcomes described by Guffon et al. (2021) in post-HSCT patients who reported an initial cognitive progress, before plateauing and then progressively declining [16]. Additionally, natural history studies show that untreated MPS I is associated with cognitive declines, with a mean DQ score between 61 and 67 after 2 years of age [21]. Eisengart et al. (2013) reported a slower rate of DQ score decline in patients with Hurler syndrome treated with ERT plus HSCT than in those treated with HSCT alone during 24 months follow-up [22]. Furthermore, Wraith et al. (2007) reported improvement in cognitive skills in patients with Hurler syndrome treated with ERT for 12 months [12]. Although the prevailing consensus has been that intravenous ERT does not cross the blood-brain barrier efficiently enough to allow any effect on the CNS, Lund et al. (2023) found that peripheral ERT is associated with a significantly lower level of cerebrospinal fluid (CSF) biomarker than that in ERT-naive patients. These findings propose that a small amount of ERT crosses the blood-brain barrier and has activity in the CSF space [23]. Despite severe or mild cognitive deficiencies at the beginning of treatment, some patients in the present cohort did not experience cognitive regression. Additionally, 73% alive patients maintained an IQ ≥ 70 after 5 to 15 years of treatment, which exceeded the results reported by Shapiro et al [24]. The results of the present study are similar to the findings of Gardin et al. (2023) reporting 69% patients with an IQ ≥ 70 at a median of 9 years post-HSCT [25].

Adenoidectomy, tonsillectomy and/or T-tube insertion interventions are common ENT procedures performed in patients with MPS I during the initial 3 years of treatment. ERT improves the auditory transmission of severe patients’ hearing loss, but it does not seem to have an impact on perception impairment. The majority (73%) of patients underwent carpal tunnel surgery before or after the initiation of enzyme therapy.

### Attenuated patient group

In patients with the attenuated MPS I, laronidase demonstrated effectiveness in improving GAGu levels. The reduction in the GAGu levels exceeded 70% after 6 months of treatment and remained stable throughout the 10-year treatment period, favouring ERT. Additionally, 65% patients maintained a standardised GAGu level reported at age. It is important to note that GAGu levels are physiologically higher in younger children < 4 years of age compared to children aged 5 to 8 years. Greater clinical severity in younger patients with attenuated MPS I is associated with markedly reduced residual alpha-L-iduronidase activity, resulting in significantly elevated GAGu levels [26]. FVC% remained stable or improved in 75% patients after 10 years of treatment in alignment with the findings from Laraway et al. (2016) that reported stabilisation of mean FVC values post ERT [2]. However, in patients with deteriorated respiratory function after 7 years of follow-up, this decline was associated with particularly elevated levels of anti-laronidase antibodies or occurred following treatment discontinuation. These findings were supported by data from Jones et al. (2020) that reported a significant and sustained reduction in GAGu excretion in patients receiving long-term ERT. Moreover, a reduction in the GAGu levels was reported to be associated with improvements in 6-minute walk test, stabilisation of pulmonary function, improved growth velocity and an improved CGI-C score [11].

All attenuated patients had valve dysplasia before starting treatment. Four patients benefited from valve replacement; three patients were diagnosed and treated late in adulthood, whereas the fourth patient initiated treatment at the age of 4.5 years, but he deteriorated probably because of a high level of anti-laronidase antibodies requiring valve replacement at the age of 18 years (the patient died during the procedure following an intubation issue). Despite late treatment for some patients, ERT slowed progression of adverse cardiac course for most patients. Stabilisation of mitral valvulopathy was observed in 66% patients and aortic valvulopathy in 63% patients after 10 years of treatment. None of the patients developed myocardiopathy. Only one patient presented with arrhythmia four years after ERT discontinuation. Laronidase had a positive impact on growth in patients who received it before 9 years of age [Z score = − 0.6 (−2) remained within normal values]. These results are consistent with findings from Laraway et al. (2016), which described superior growth in attenuated patients treated before the age of 10 years. Moreover, patients treated before 4 years of age had the height-for-age Z-scores closer to the normal range throughout the follow-up [2]. Findings from the Polgreen et al. (2022) registry study also suggest better height outcomes in ERT-treated patients than the natural disease history in patients with attenuated MPS I, with the estimated average height Z-scores in the ERT-treated patients and natural history period at 10 years of age being − 2.4 for females and − 1.4 in males versus − 3.3 in females and − 2.9 in males, respectively [27]. Viskochil et al. (2019) also reported similar findings in untreated patients with the estimated median height of untreated individuals with attenuated MPS I declining below the third percentile by 9 years of age and continuing to decline through 18 years of age (decline appeared to be slow in males at an earlier age than in females) [17].

Cognitively, 84% attenuated patients had normal cognitive development, and none deteriorated during the follow-up on treatment. These results were superior to those reported by Shapiro et al. (2015), which described a loss of 1.6 points per year in attenuated patients [24]. Initiating ERT early can substantially improve the overall health, cardiorespiratory symptoms and surgical outcomes for orthopaedic complications. This approach helps maintain mobility, autonomy and an independent quality of life. ERT effectively reduced the occurrence of SAS in the majority (68%) of patients and enhanced hearing loss transmission in attenuated patients. However, it had no impact on perception impairment. The improvement in transmissional impairment had a positive impact on language development and allowed for better tolerance of hearing aids.

## Conclusion

Several patients with severe MPS I have evolved remarkably in terms of respiratory, cardiac function, autonomy of daily activities and cognition under ERT, with significant decrease of GAGu levels. ERT with laronidase is effective in some patients with severe MPS I (Hurler syndrome). The beneficial effect of laronidase on the cognitive function of severe patients may be linked to its efficacy in improving cardiorespiratory and ENT somatic functions. Laronidase is effective in slowing the disease progression regarding cardiorespiratory function, ENT and the statural growth of attenuated patients. Early treatment and regular monitoring of attenuated patients along with a multidisciplinary management approach (including biological, cardiac, ENT and orthopaedic management) help preserve their cognitive ability and autonomy to maintain an optimal quality of life.

## Electronic supplementary material

Below is the link to the electronic supplementary material.

Supplementary Material 1

## Data Availability

The data that support the findings of this study are available from the corresponding author on request. The data are not publicly available due to privacy or ethical restrictions.

## Abbreviations

DQ: Developmental quotient
ECG: Electrocardiography
ENMG: Electromyoneurograph
ENT: Ear nose and throat
ERT: Enzyme replacement therapy
FVC: Forced vital capacity
GAGu: Urinary glycosaminoglycans
HSCT: Haematopoietic stem cell transplantation
IDUA: α-L-iduronidase
IQ: Intellectual quotient
MPS: Mucopolysaccharidosis
SAS: Sleep apnoea syndrome
WAIS: Wechsler Adult Intelligence Scale
WISC: Wechsler Intelligence Scale for children
