# Supplementary material for: Clinical outcomes of exclusive enzyme therapy (laronidase) in a cohort of patients with mucopolysaccharidosis type I
Source: Orphanet J Rare Dis. 2025 Dec 6;21:11. doi: 10.1186/s13023-025-04157-6 (PMC12797645; doi:10.1186/s13023-025-04157-6)
Supplement: Supplementary file 1 — Supplementary Material 1 [file 13023_2025_4157_MOESM1_ESM.docx]

**Supplementary Figure 1. Evolution of growth in A) severe and B) attenuated patients with MPS I by subgroup**


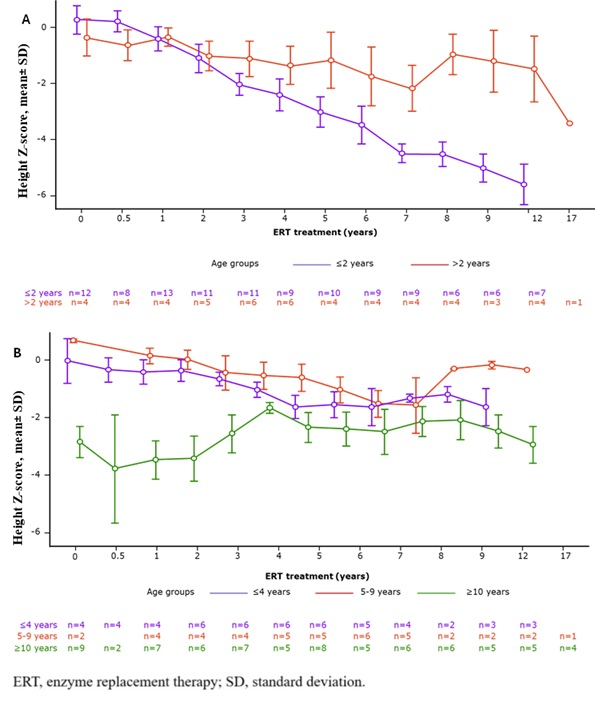


**
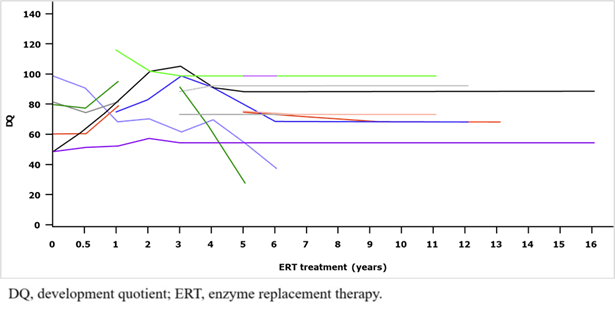
 Supplementary Figure 2: Evolution of DQ per patient post treatment with laronidase**

**Supplementary Table 1: The limits of normal GAGu values by age with the harmine method**

| **Age** | **μg glucuronic acid/mg creatinine**  **(5th–95th percentile)** |
| --- | --- |
| 0–3 weeks | 22–80 |
| 3 weeks–6 months | 12–56 |
| 6 months–1 year | 9–46 |
| 1 year–3 years | 8–29 |
| 3 years–7 years | 6–23 |
| 7 years–15 years | 3–16 |
| 15 years–20 years | 1–15 |
| Adults | 1–8 |
| GAGu, urinary glycosaminoglycans. | |

**Supplementary Table 2: Scores of autonomy of daily activities, mobility, competency and assistance score for severe MPS I**

| **Patients with attenuated MPS I** | **Current age (years)** | **Age at ERT initiation** | **Autonomy score (personal care)** | **Autonomy skill score %  (personal care)** | **Mobility score** | **Mobility skill score (%)** | **Assistance score (max score: 39)** |
| --- | --- | --- | --- | --- | --- | --- | --- |
| **Patients treated before 2 years of age** | | | | | | | |
| 1 | 16 | 1.1 | 0.74 | 100 | 1.4 | 100 | 0 |
| 2 | 13 | 1 | Impossible tasks | 0 | Impossible tasks | 0 | 39 |
| 3 | 12 | 0.33 | 2.04 | 78 | 1.4 | 100 | 4 |
| 4 | 12 | 0.67 | 2.44 | 63 | 8 | 100 | 27 |
| 5 | 8 | 1.58 | 2.48 | 33 | 0.3 | 100 | 16 |
| 6 | 6 | 0 | 1.85 | 92 | 0.4 | 100 | 3 |
| **Patients treated after 2 years of age** | | | | | | | |
| 7 | 19 | 2.5 | 3.78 | 3.70 | 6 | 40 | 30 |
| 8 | 14 | 3.1 | 1.03 | 100 | 0 | 100 | 0 |
| 9 | 12 | 2.25 | 0 | 100 | 0 | 100 | 0 |
| 10 | 12 | 2.17 | 1.9 | 100 | 2.9 | 100 | 3 |
| 11 | 9 | 3.1 | 1.28 | 100 | 0.7 | 100 | 6 |
| MPS-HAQ self-questionnaire is a 52-question tool that covers the area of autonomy (personal care), reflection of daily activities, the field of mobility and the extent of assistance required from the caregiver in the exercise of these activities. For the scores of the autonomy/personal care and mobility domains, scores ranged from 0 to 10, with lower score corresponding to better overall status of the patient. Competence scores ranged from 0 to 100 with higher scores corresponding to better status. Support scores ranged from 0 to 39 with lower scores corresponding to the lower need for caregiver assistance. Patient 2 and Patient 7 required major or total assistance, Patient 4 and Patient 5 required moderate assistance, the other patients mentioned required no assistance.  ERT, enzyme replacement therapy; MPS, mucopolysaccharidosis type I; MPS-HAQ, MPS Health Assessment Questionnaire. | | | | | | | |

**Supplementary Table 3. Scores of autonomy of daily activities, mobility, competency and assistance score for attenuated MPS I**

| **Patients with attenuated MPS I** | **Current age (years)** | **Age at ERT initiation** | **Autonomy score (personal care)** | **Autonomy skill score % (personal care)** | **Mobility score** | **Mobility skill score (%)** | **Assistance score (max score: 39)** |
| --- | --- | --- | --- | --- | --- | --- | --- |
| **Patients treated after the age of 10 years** | | | | | | | |
| 1 | 49 | 36 | 4.67 | 11 | Impossible task | 0 | 36 |
| 2 | 49 | 34 | 3.44 | 100 | 3.4 | 100 | 3 |
| 3 | 46 | 30 | 0.8 | 92 | 3.5 | 100 | 4 |
| 4 | 42 | 24 | 0.7 | 100 | 2.2 | 100 | 1 |
| 5 | 39 | 22 | 4.2 | 100 | 6.9 | 100 | 15 |
| 6 | 23 | 11 | 0 | 100 | 0 | 100 | 0 |
| 7 | 18 | 14 | 0.52 | 100 | 0.7 | 100 | 4 |
| **Patients treated between the ages of 5 and 9 years** | | | | | | | |
| 8 | 23 | 6 | 0 | 100 | 0 | 100 | 0 |
| 9 | 19 | 8 | 1.37 | 100 | 1.9 | 100 | 7 |
| 10 | 17 | 9 | 2.3 | 100 | 3.8 | 100 | 9 |
| 11 | 15 | 4 | 0 | 100 | 0 | 100 | 0 |
| **Patients treated before the age of 4 years** | | | | | | | |
| 12 | 17 | 3 | 0.44 | 100 | 3.5 | 100 | 5 |
| 13 | 15 | 4 | 0.63 | 100 | 0.2 | 100 | 0 |
| 14 | 14 | 3 | 0.92 | 100 | 0.5 | 100 | 1 |
| 15 | 10 | 4 | 0.15 | 100 | 0 | 100 | 0 |
| 16 | 9 | 1.5 | 1.3 | 100 | 0.8 | 100 | 6 |
| Patient 1 required major or total assistance, Patient 5 required moderate assistance, the other patients mentioned required no assistance.  ERT, enzyme replacement therapy; MPS, mucopolysaccharidosis type I; MPS-HAQ, MPS Health Assessment Questionnaire. | | | | | | | |
